# The Effect of Glycol Derivatives on the Properties of Bio-Based Unsaturated Polyesters

**DOI:** 10.3390/polym14152970

**Published:** 2022-07-22

**Authors:** Olga Pantic, Milica Spasojevic, Enis Dzunuzovic, Marija S. Nikolic, Sanja Savic, Maja Markovic, Pavle Spasojevic

**Affiliations:** 1 Institute of Chemistry, Technology and Metallurgy, Center of Excellence in Environmental Chemistry and Engineering, University of Belgrade, Njegoševa 12, 11000 Belgrade, Serbia; olga.pantic@ihtm.bg.ac.rs (O.P.); sseslija@tmf.bg.ac.rs (S.S.); 2 Innovative Centre of Faculty of Chemistry, University of Belgrade, Studentski Trg 12–16, 11000 Belgrade, Serbia; smilica84@gmail.com; 3 Faculty of Technology and Metallurgy, University of Belgrade, Karnegijeva 4, 11000 Belgrade, Serbia; edzunuzovic@tmf.bg.ac.rs (E.D.); mnikolic@tmf.bg.ac.rs (M.S.N.); 4 Innovation Center of Faculty of Technology and Metallurgy, University of Belgrade, Karnegijeva 4, 11000 Belgrade, Serbia; mmarkovic@tmf.bg.ac.rs; 5 Faculty of Technical Sciences, University of Kragujevac, Svetog Save 65, 32000 Cacak, Serbia

**Keywords:** bio-based unsaturated polyester resins, glycol, dimethyl itaconate, thermal properties, thermomechanical properties, mechanical properties

## Abstract

The scope of the present study was to prepare fully bio-based unsaturated polyester resins (UPRs) with comparable properties to the commercial formulations. The focus was set on the determination of the optimal prepolymer formulation using the same set of diacids (itaconic and succinic acid) and different diols (propylene glycol, isosorbide and neopentyl glycol) or its equimolar mixtures, keeping the fixed molar ratio of 1:1:2.1 in all feed compositions. Instead of commonly used styrene, bio-based dimethyl itaconate was used as a reactive diluent (RD). The rheology of the obtained resins was studied in detail. The effect of the used diol on structural (FTIR), thermal (DSC), thermomechanical (DMA), and mechanical (tensile) properties was explained. The properties of UPRs were found to be highly dependent on the diol used in the prepolymer formulation. The UPR with an equimolar ratio of propylene glycol and neopentyl glycol was shown to be the most promising candidate to compete with the commercial petroleum-based resins.

## 1. Introduction

Polyesters are one of the leading polymer materials with versatile properties that have found a variety of applications in the automotive industry, plastics industry, biomedical field, etc. [1,2,3]. Among all polyester materials, unsaturated polyesters (UPs), characterized by low molecular weight and high polydispersity, have attracted significant attention from many research groups; namely, the presence of a double bond in the polymer chain allows for curing with unsaturated monomers and, thus, producing thermosetting polyesters known as unsaturated polyester resins (UPRs). The properties of UPRs, such as viscosity, processability, thermal and mechanical properties, can be tailored by careful choice of the diols and diacids, used to prepare prepolymers (UPs) and unsaturated monomers as reactive diluents (RDs), responsible for cross-linking [1,4,5]. By varying the ratio between the starting compounds, UPRs with various properties can be obtained. Moreover, the characteristics of the UPRs also depend on the extent of the cure reaction, indicating the importance of understanding the curing process [1]. Thus, under appropriate reaction conditions, UPRs with great mechanical properties, high chemical resistance, facile processing and a low cost have been obtained and widely applied in building and construction, automotive, marine, electrical, decorative and aerospace industries [6,7].

Most commercially available UPRs are of petrochemical origin, which represents their main drawback from an economic and environmental point of view [6]. Petrochemical compounds are usually toxic and volatile, and thus require careful handling and disposal. Furthermore, the most common reactive diluent is styrene, known as a hazardous air pollutant, carcinogen, neurotoxin, and respiratory tract irritant [8,9]. Considering these facts, as well as the limited availability of petrochemical sources and oscillations in the price of crude oil, a great deal of attention has been focused on developing UPRs from more environmentally friendly compounds obtained from renewable sources. Renewable compounds would also extensively reduce waste generation and emissions, and thus contribute to future sustainability.

Some research groups have used bio-based compounds, such as soybean oil, maleic anhydride, pentaerythritol, isosorbide, ethylene glycol, adipic, suberic and sebacic acid, to prepare UPRs with comparable or even more improved properties compared to commercial UPRs [10,11,12]. However, the obtained UPRs were not completely “green” resins, since the cross-linked structure was acquired with styrene as RD.

The replacement of styrene with bio-based compounds has been challenging, since the resins obtained with “green” substitutes suffered from increased resin viscosities, low glass transition temperature, low elastic and storage modulus or bad processability [13,14,15,16,17,18,19]. La Scala and coworkers substituted styrene with a fatty acid-based monomer, methacrylated fatty acid. The vinyl ester resin obtained with this unsaturated monomer showed substantially higher viscosity and considerably lower Tg. A decrease in the size of the fatty acid slightly improved both the polymer properties and viscosity. However, the acceptable flexural, fracture and thermo-mechanical properties and resin viscosity were obtained by including styrene in the resin formulation (10–20 wt.% styrene and methacrylated lauric acid as primary RD) [13]. Campanella et al. prepared resins using an acrylated fatty acid methyl ester-based monomer as RD, and used either acrylated epoxidized soybean oil, maleinated soybean oil monoglyceride or maleinated castor oil monoglyceride as a cross-linker. They have also shown that resin properties comparable to those of commercial resins can only be achieved by incorporating a certain amount of styrene in the resin. Five percent of butyrated kraft lignin improved the mechanical properties of the resin, but impaired its viscosity [14,15]. Polymer films synthetized by oxidative copolymerization of tannin linoleate/acetate mixed esters with linseed oil and tung oil had ambient modulus values between 0.12 and 1.6 GPa, with glass transition temperatures in the range of 32 to 72 °C, lower than that of commercial resins [16].

Besides fatty acid monomers, lignin-based aromatic model compounds have also been used as styrene substitutes [17,18]. Methacrylated guaiacol and methacrylated eugenol were used as RDs in vinyl ester resins to provide resins with high Tg values (127 and 153 °C). However, the resin viscosities were substantially higher than those of vinyl ester–styrene blends (937 and 1148 cP in comparison to 16.4 cP) [17]. Resins prepared with a methacrylated lignin-based bio-oil, consisting of methacrylated phenol, guaiacols, and catechols, have shown high storage moduli and Tg values (≥2.5 GPa and ≥115 °C), but suffered from very high resin viscosity [18]. 

Bio-based vinyl levulinate has been another potential candidate for styrene replacement in the preparation of UPRs. However, 5.5 wt.% of vinyl levulinate monomer remained unpolymerized due to the unfavorable reactivity ratio. It resided in the resin network and behaved as a plasticizer, which resulted in lower both relaxation and elastic moduli at the rubbery plateau, and worse mechanical properties of the resin [19].

To overcome viscosity issues, some research groups have focused on the preparation of UPRs from systems composed of oligomers and monomers. The obtained resins had low storage moduli and glass transition temperature, and high elongation at break [20,21,22,23].

In order to prepare fully bio-based UPRs with properties comparable to those of commercial UPRs and with no issues related to its viscosity and thus processability, we synthetized UPRs from two diacids (itaconic and succinic acid), different diols (propylene glycol, isosorbide and neopentyl glycol) and dimethyl itaconate as RD. Itaconic and succinic acid are good polyester building blocks that can be easily obtained from biomass [20,21,24]. Itaconic acid was first obtained from the thermal decomposition of citric acid in 1837 by Baup [25]. Later, this acid was prepared by the fermentation of glucose or other sugars using various yeasts or filamentous fungi [26]. Succinic acid was first produced by amber distillation in 1546. It can be obtained during the process of fermentation of cornstarch, cane molasses, whey, wood hydrolysates and glycerol by the action of particular strains of bacteria [27]. Bio-based propylene glycol was synthetized from glycerin and hydrogen by hydrogenolysis, whereas glycerin was prepared under anaerobic conditions by converting monosaccharides with S. cerevisiae into ethanol and glycerin as a by-product, or by the action of bacteria and algea [27,28]. Isosorbide is obtained by the sequential dehydration of sorbitol, which is a product of the fermentation of sucrose or glucose–fructose mixtures using Zymomonas mobilis, or the enzymatic hydrolysis of cereal-extracted starch and mannans [28,29]. BASF reported the production of biomass balanced neopentyl glycol, whereas Perstorp Group produced neopentyl glycol with up to 50 percent bio-based content [30,31]. Joo and coworkers prepared dimethyl itaconate by the lipase-catalyzed esterification of itaconic acid, obtained from rice wine waste using the engineered C. glutamicum strain [32].

This study aimed to prepare fully bio-based unsaturated polyester resins with appropriate mechanical properties and processability, which would allow for a wide range of applications and the complete replacement of commercial petroleum-based UPRs. To this end, unsaturated polyesters were prepared with different polyols (propylene glycol, isosorbide and neopentyl glycol) or their mixture, and subsequently cross-linked in the presence of dimethyl itaconate as RD. UPRs with an equimolar ratio of propylene glycol and neopentyl glycol, cross-linked at 60 °C, have shown good mechanical properties and a great potential to substitute petroleum-based resins.

## 2. Materials and Methods

### 2.1. Materials 

The itaconic acid (99%) (IA), succinic acid (99%) (SA), propylene glycol (99%) (PG), neopentyl glycol (99%) (NPG), isosorbide (98%) (IS), methyl ethyl ketone peroxide (MEKPO), cobalt octoate, hydroquinone (pro analysis) (HQ), ethanol (99%) and toluene (99%) were supplied from Acros Organics, Geel, Belgium. Isopropanol and tetrahydrofuran were purchased from Macron Fine Chemicals, Center Valley, Pennsylvania, USA, and Fisher Chemical, Waltham, MA, USA, respectively. Zinc acetate (99.99%) (ZnAc) and dimethyl itaconate (99%) (DMI) were purchased from Sigma-Aldrich, St. Louis, MI, USA. All chemicals were used as received.

### 2.2. Preparation

Prepolymer was synthesized by the reaction of melt polycondensation with diacids and diol (propylene glycol, neopentyl glycol or isosorbide). The molar ratio of itaconic acid, succinic acid and diol was 1:1:2.1, respectively. Toluene (0.5 wt.% based on monomers) was used to increase the rate of water removal, and hydroquinone (150 ppm) was chosen as a free radical scavenger. Zinc acetate was added as a catalyst of the esterification reaction (25 wt.% in respect to the total weight of acids). The compounds were mixed and the reaction was carried out in an open system consisting of a three-neck round-bottom flask, stirrer, Dean–Stark and thermometer in an inert atmosphere. The temperature of the reaction mixture was changed within the range of 110 to 190 °C, and raised by 10 °C per hour. The reaction was carried out until the acid value reached 50, except for the synthesis using isosorbide, where the acid value reached 71, after which the reaction was stopped due to the high viscosity of the reaction mixture. The resin was cooled to 90 °C in an inert atmosphere and vacuumed. Subsequently, prepolymer was dissolved in the reactive diluent dimethyl itaconate (30% *w/w*). For curing of the resins, the initiator methyl ethyl ketone peroxide (2.5% *w/w*) and activator cobalt octoate (1% *w/w*) were added, and the obtained mixture was homogenized and poured into Teflon molds. The Teflon molds were placed in an air oven and left for 24 h at 60 °C. Additionally, the samples were kept at 120 °C for one hour to harden. 

The acid value (AV) was defined as the number of milligrams of KOH needed to neutralize 1 g of resin and was determined according to ASTM D465-01. Around 0.5 g of resin was titrated with a KOH solution in ethanol (0.1 mol/L). The AV is calculated using the following equation:

(1) $$AV=56.1\;\cdot \;c\left(KOH\right)\;\cdot \;V\left(KOH\right)/W$$

where c(KOH) and V(KOH) are concentration (mol/L) and volume (L) of the used titrant, respectively, and W is weight of the resin being examined (g). 

The samples are nominated as UPR-PG, UPR-NPG and UPR-IS, depending on the diol type (propylene glycol, neopentyl glycol or isosorbide) or its equimolar mixture UPR-PG/IS, UPR-PG/NPG, UPR-NPG/IS applied for the feed compositions. 

### 2.3. Methods

The Fourier transform infrared (FTIR) spectra of powdered samples were recorded in transmittance mode for the wavelength range of 600–4000 cm^−1^ with a resolution of 4 cm^−1^, using a Nicolet™ iS10 FTIR spectrometer.

The glass transition temperature (Tg) of the samples was determined by a differential scanning calorimeter—DSC (DSC-60Plus differential scanning calorimeter, Shimadzu). The weight of the samples was limited to 5 ± 0.5 mg. The samples were first heated from –20 °C to 200 °C at a rate of 10°C/min under a nitrogen purge gas flow of 50 mL min^−1^. Then, the second heating run was carried out under the same conditions. The Tg of the samples was determined from the second heating run.

Shear rate viscosity dependences of UPR samples were recorded on a Discovery Hybrid Rheometer HR2 (TA Instruments). Parallel plate geometry was used with a plate diameter of 25 mm (gap 0.5 mm) and with a Peltier system for temperature control. Rheological data were collected in the shear rate range of 0.1–100 s^−1^ (with 5 points per decade) at temperatures in the range of 25–55 °C (step, 10 °C). Averaging and equilibration times at each shear rate point were set at 10 s.

The gel content was calculated by extraction in tetrahydrofuran (THF). The cured samples were cut into rectangular shapes, with dimensions of 40 × 10 × 4 mm, and their weight was determined (Wi). The prepared samples were immersed into THF for 28 days at room temperature. Insoluble fractions corresponding to the inflated polymer network were filtered, carefully dried under vacuum, and then measured (Wsol). The gel content was calculated as follows:

(2) $$GC\;\left(\%\right)\;=\left(Wsol⁄Wi\;\right)\;\times 100$$

The uniaxial tensile mechanical properties of the investigated UPR samples were evaluated using the Shimadzu Autograph AGS-X servo-hydraulic testing machine, equipped with a 1 kN load cell at ambient temperature according to ASTM D638. Four measurements were performed for each UPR sample at a testing rate of 2 mm/min. The average values of the break stress and break stroke strain, the standard deviations and Young’s modulus were determined. Young’s modulus was calculated by the software TRAPEZIUM X from the linear part of the stress–strain curve.

The dynamic mechanical properties of the samples were analyzed by a Discovery HR-2 (TA Instruments, New Castle, DE, USA). The prism-shaped samples rectangular bars (60 × 12 × 2 mm) were exposed to the fixed strain amplitude of 0.1% and angular frequency of 1 Hz in the temperature range of 25 to 150 °C, changing stepwise (with a step of 2.5 °C). The storage modulus (G′/GPa), loss modulus (G″/MPa) and damping factor (tan δ) were the measured data, whereas the glass transition temperature (Tg) was determined as the temperature where tan δ reached the maximum value. All results were obtained from the second heating cycle.

## 3. Results and Discussion

**Scheme 1:**
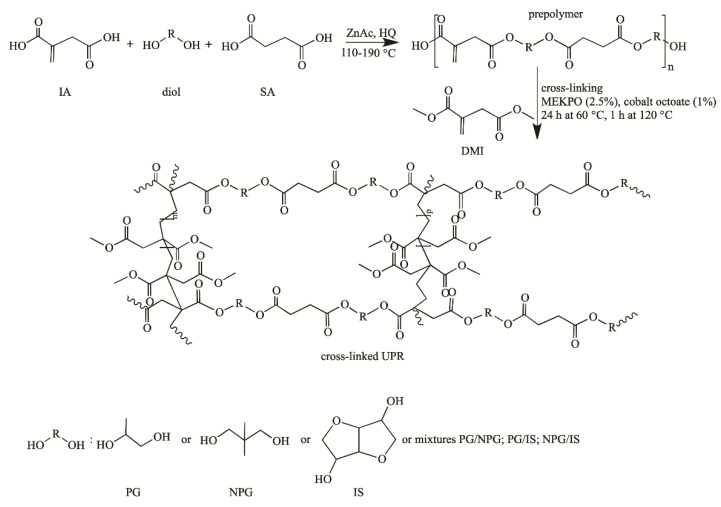
General mechanism of the polycondensation reaction and cross-linking.

The effect of glycol derivatives on the properties of bio-based unsaturated polyester resins was investigated. The prepolymer was synthesized via melt polycondensation and was diluted by dimethyl itaconate. The synthesis and cross-linking pathways are presented in Scheme 1.

**Figure 1:**
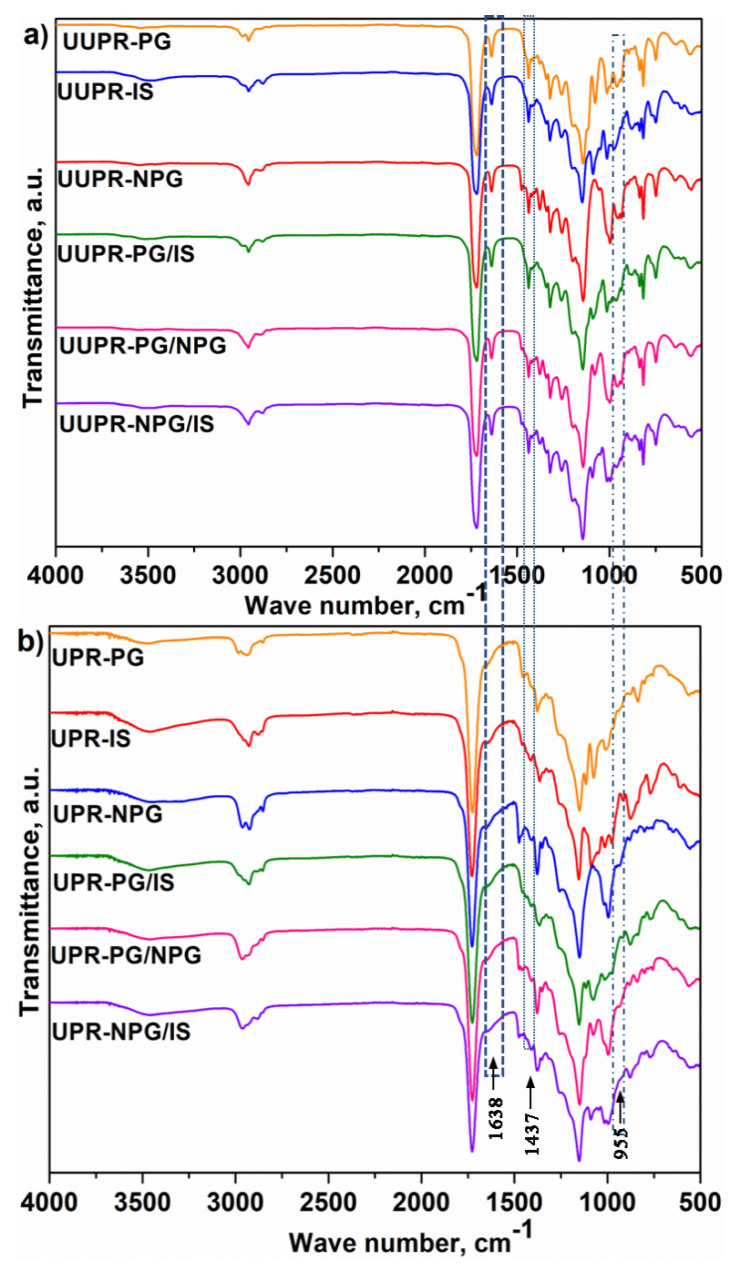
FTIR spectra of: (**a**) the uncured and (**b**) cured samples.

The chemical structure of both uncured (UUPR) and cured (UPR) resins was analyzed by FTIR spectroscopy, and the corresponding spectra are shown in Figure 1. The spectra of both uncured and cured UPR samples showed a band at about 3490 cm^−1^, originating from OH stretching vibration. The bands corresponding to asymmetric and symmetric stretching vibrations of the aliphatic C-H group are observed in the range of 2990 to 2850 cm^−1^. The bands at 1720 cm^−1^ correspond to the ester carbonyl stretching vibrations. The other characteristic band at 1150 cm^−1^ can be assigned to C(=O)-O stretching vibration from the ester group. In the spectra of uncured UPR samples, the bands that can be observed at 1638 and 1437 cm^−1^ correspond to the stretching of C=C bonds and in-plane bending of vinyl C-H bonds, respectively, while the bands at 955 and 815 cm^−1^ are ascribed to out-of-plane bending of vinyl C-H bonds. In the spectra of cured UPR samples, the intensity of the bands at 1638, 1437, 955 and 815 cm^−1^ drastically decreased or completely disappeared. This indicates low or zero amounts of unreacted double bonds in cured UPR samples.

**Figure 2:**
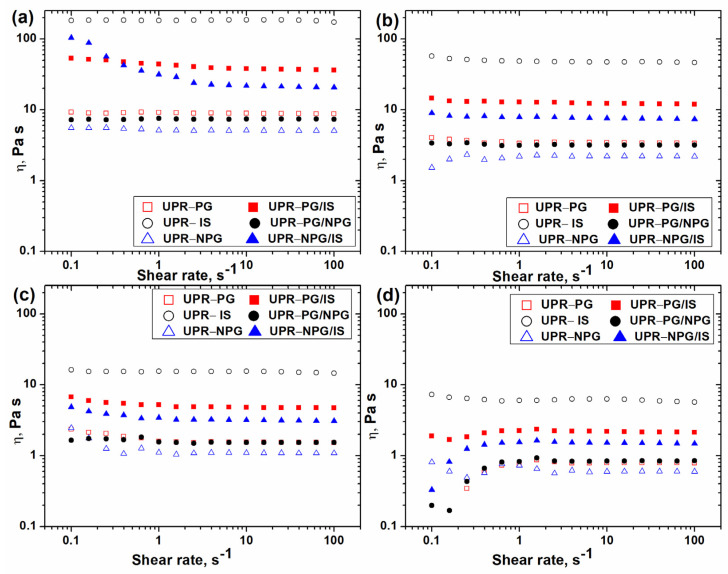
The viscosity as a function of the shear rate of the examined UP resins measured at: (**a**) 25 °C; (**b**) 35 °C; (**c**) 45 °C; and (**d**) 55 °C.

The viscosity of six examined UPR samples was measured at four different temperatures (25, 35, 45 and 55 °C) using the shear rate sweep mode. The obtained results are seen in Figure 2a–d. It is shown that the viscosity of UPRs is independent of the shear rate. This behavior is characteristic for Newtonian liquids and indicates an absence of chain entanglements. In addition, the viscosity decreases with temperature increase, and the UPR-IS sample has the highest viscosity at all temperatures. The synthesis of this UP was performed using isosorbide as the only glycol component. The introduction of an isosorbide moiety in the backbone increases its stiffness, reduces its mobility and causes an increase in the resin viscosity. Using propylene glycol or neopentyl glycol instead of isosorbide for the synthesis of UP, the flexibility and mobility of the obtained UP molecules are much larger. Therefore, the samples UPR-PG and UPR-NPG have around 20 and 37 times lower viscosity than the UPR-IS sample at 25 °C, respectively, and almost 8 and over 10 times lower viscosity at 55 °C, respectively. 

It is well known that propylene glycol is the most used glycol for UPR production, and that resins with improved heat and chemical resistance can be obtained using neopentyl glycol or hydrogenated bisphenol-A. Hydrogenated bisphenol-A can be replaced with isosorbide, which is obtainable from renewable sources. However, the main problem with UPs based on isosorbide is excessive viscosity. In order to obtain UPRs with good properties and appropriate viscosity that is acceptable for practical applications, UPs were synthesized using equimolar mixtures of isosorbide and propylene glycol, and isosorbide and neopentyl glycol as the glycol component. The incorporation of propylene glycol or a neopentyl glycol moiety in UP molecules significantly decreases the viscosity of UPRs. Thus, the UPR-PG/IS and UPR-NPG/IS samples have almost five times and over eight times lower viscosity than the UPR-IS sample at 25 °C, respectively, and almost three times and over four times lower viscosity at 55 °C, respectively. This behavior is a result of the increased flexibility and mobility of the UP chains after the incorporation of propylene glycol and a neopentyl glycol moiety in their structure. The effect is more pronounced by the incorporation of a neopentyl glycol moiety, because the polarity of the UP molecules is more reduced by the incorporation of a neopentyl glycol moiety. This leads to a decrease in intermolecular interactions between UP molecules and causes a drop in the viscosity of the UPRs.

**Figure 3:**
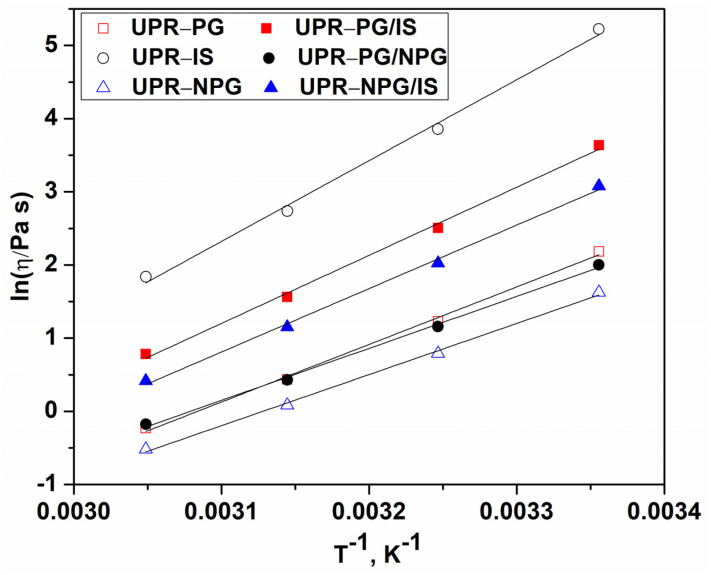
Plots of ln η as a function of 1/T at a constant shear rate (10 s^−1^).

The effect of the composition of UPs on the viscosity of UPRs can be observed through the values of activation energy of flow at a constant shear rate (Ea_η_), which were calculated for all the examined UPR samples from the slope of the obtained linear dependence of the viscosity logarithm on the reciprocal temperature at the shear rate of 10 s^−1^ (Figure 3). Table 1 shows that the obtained Ea_η_ values for the UPR-PG/IS and UPR-NPG/IS samples are lower than the value for the UPR-IS sample, indicating that the incorporation of propylene glycol and a neopentyl glycol moiety in the UP molecules enhances their flexibility and mobility and also decreases their polarity. This leads to a reduction in intermolecular interactions and viscosity.

The curing of unsaturated polyesters is an important feature because it governs the applicative potential. The cure reaction occurs through the complex free radical mechanism. Complexity is a consequence of the presence of two co-monomers (DMI and prepolymer) and rapid changes in the system rheology; namely, copolymerization leads to the early formation of microgels, even at conversion levels as low as 3–4% [33,34]. Subsequently, the exothermic reaction results in an increase in temperature and a rapid increase in the system viscosity, which can omit good cross-linking efficiency. In order to evaluate the cross-linking efficiency, the gel content of cross-linked samples was determined after immersion in THF for 28 days. The obtained results are given in Table 2. The samples where PG and NPG are used as a glycol component show high values of gel content (>97%), whereas reduced values are obtained for the samples with IS. This is especially evident when IS is used as a sole glycol component. In this case, the gel content value is very low (75.49%), indicating low cross-linking density. The cross-linking reaction mainly depends on the prepolymer and reactive diluent reactivity. Considering DMI was used as a reactive diluent in each sample, and that unsaturation in each prepolymer originates from the itaconate moiety, it could be concluded that the prepolymer structure caused different curing; namely, very rigid isosorbide bicyclic rings significantly declined the prepolymer chain mobility, thus its reactivity and also overall cross-linking.

**Figure 4:**
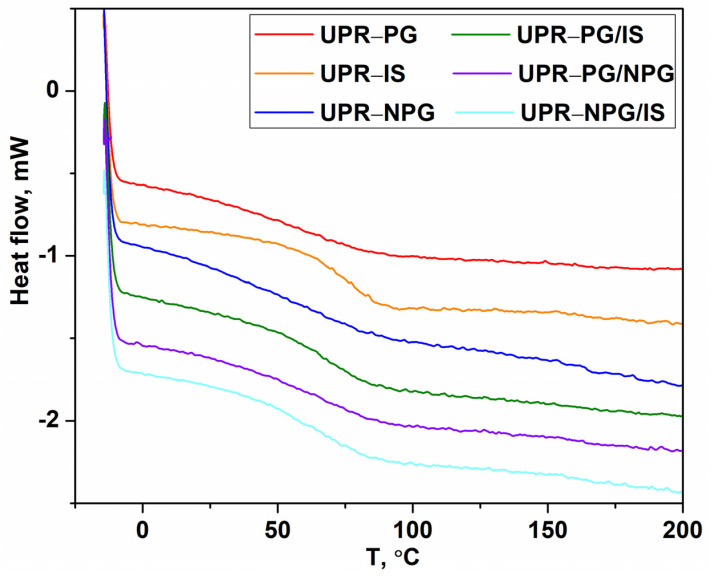
DSC thermograms of cured samples.

The DSC analysis was used to determine the thermal characteristics of the cross-linked samples. The glass transition temperature Tg is the temperature at which a polymeric material changes from a glass-like state to a soft rubbery state. The Tg depends on the polymer chain molar weight and mobility, as well as the cross-linking density [2,20]. The DSC thermograms of cured samples (second run) are shown in Figure 4. The Tg values are determined from the midpoint of the heat capacity change from the second cycle. The obtained values are similar and in the range of 63.7 to 70.8 °C. Considering that the cross-link density is a governing factor for the Tg of unsaturated polyesters, it can be assumed that all samples have a similar cross-link density. However, the results of gel content show that the UPR-IS samples have the lowest value of gel content, but the highest value of Tg, which is determined by DSC. This could be a consequence of the incorporation of very rigid isosorbide bicyclic rings that significantly hinder the chain mobility and, thus, increase the Tg values. Similarly, UPR-PG/IS has a lower value of gel content compared to other samples, but a similar value of Tg (Table 2).

**Figure 5:**
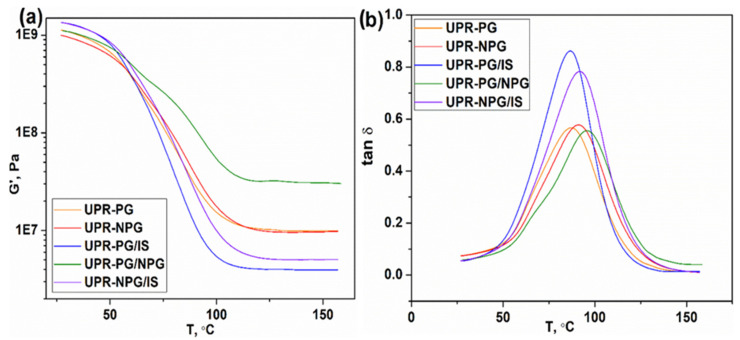
DMA curves of the storage modulus, G′ (**a**) and loss factor, tan δ (**b**) of the prepared UPRs at a fixed strain amplitude of 0.1% and a single angular frequency of 1 Hz.

The dynamic mechanical analysis was used to investigate the thermomechanical properties of the prepared resins. The plots of storage modulus (G′) and loss factor (tan δ) as a function of temperature are presented in Figure 5a,b, respectively. Due to the high brittleness and low strength of UPR-IS, this sample could not be analyzed by this method. The peak of the tan δ was used to determine the Tg values of the resins, whereas the cross-linking density was calculated from the theory of rubbery elasticity, as shown in Equation (3):

(3) $$\nu_{e}=G^{\prime }/\left(3\times R\times T\right)$$

where G′ is the storage modulus taken from the rubbery plateau region, R is the gas constant, and T is the corresponding absolute temperature in the rubbery plateau region. The values of the storage modulus at 25 °C (G′_25°C_), storage modulus at 135 °C (G′_135 °C_), glass transition temperature, obtained as a peak of the tan δ-T curve (Tg _DMA_), and calculated values of the crosslinking density (ν_e_) are presented in Table 2.

When considering unsaturated polyester resins, the glass transition temperature and storage modulus are mainly directed by the cross-linking density and rigidity of the macromolecule chain segments. At lower temperatures, when the material is in a glassy state, the increase in cross-linking density and chain rigidity leads to the increase in Tg and storage modulus. With an increasing temperature, the thermosets undergo the glass transition to a rubbery state, presented by a dramatic decrease in G′ (Figure 5a). Here, G′ values at the glassy state vary from 0.996 GPa to 1.35 GPa. The incorporation of rigid isosorbide bicyclic rings results in the increase in G′ values. These samples show lower values of cross-linking density calculated from the theory of rubbery elasticity (Table 2). This indicates that the isosorbide bicyclic rings rigidity exhibits a very pronounced effect on the material macroscopic properties. It is interesting to note that the sample UPR-PG/NPG shows the second lowest value of G′ at the glassy state, but by far the highest values of G′ at the rubbery state. This is attributed to the higher flexibility of the polymer chains, which is a consequence of the higher flexibility of neopentyl glycol compared to propylene glycol and isosorbide. The similar shape of tan δ curves (Figure 5b) indicates comparable homogeneity of the formed networks in all samples. In addition, the tan δ curves of samples without IS show broader peaks, implying their better damping capacity. 

**Figure 6:**
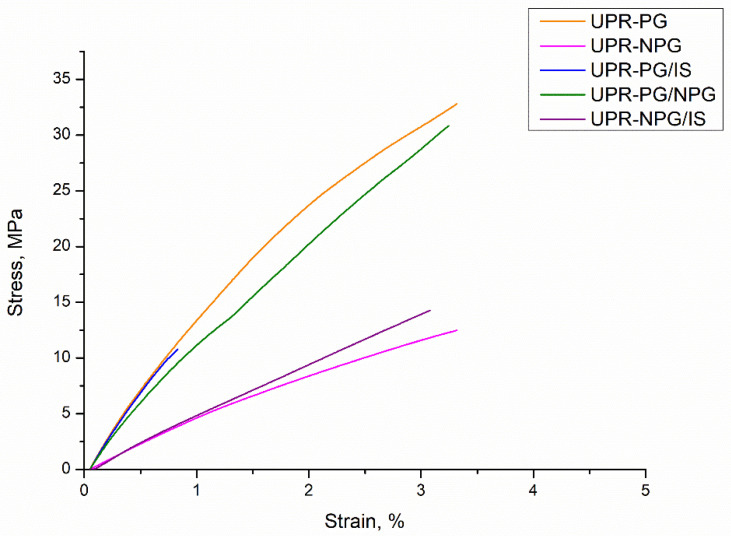
Stress–strain dependency for uniaxial tensile testing.

The mechanical properties of prepared resins were investigated by uniaxial tensile testing. The testing results are presented in Figure 6 by plotting stress–strain curves. Here as well, it was not possible to measure the sample UPR-IS due to the breakage. The obtained values of the Young’s modulus, the ultimate strength and elongation at break are shown in Table 3. The samples prepared with isosorbide exhibit the highest values of Young’s modulus, but the lowest values of ultimate strength and elongation at break. This is a consequence of the high rigidity of isosorbide bicyclic rings and low cross-linking density. On the other hand, the samples prepared from neopentyl glycol and propylene glycol have slightly lower values of Young’s modulus, but significantly higher values of ultimate strength and elongation at break. The sample UPR-PG/NPG shows the highest values of ultimate strength and elongation at break.

## 4. Conclusions 

The present study reports the influence of the specific polyols used for the synthesis of the prepolymer (based on itaconic and succinic acid) on the comercially valuable UPRs properties. The structure of the uncured and cured resins was investigated by FTIR, confirming sucessful synthesis. The effect of prepolymer composition on the viscosity of UPRs was thoroughly investigated. The UPR-IS showed the highest viscosity, which makes this formulation unfavourable for commercial applications. By substituting isosorbide with propylene glycol or neopentyl glycol in the UP synthesis, the obtained samples showed lower viscosity values. Samples where propylene glycol and neopentyl glycol were used in a prepolymer formulation showed the highest value of gel content (>97%), while samples with isosorbide showed lower gel content, indicating poor cross-linking density. All the samples showed similar Tg values in the range of 63.7 to 70.8 °C. The Young’s modulus, ultimate strength and elongation at break were found to be in the range of 1.23 to 1.74 GPa, 12.5 to 35.1 MPa and 0.90 to 4.39%, respectively. UPRs with the equimolar ratio of propylene glycol and neopentyl glycol cured at 60 °C have shown good mechanical properties and great potential to substitute petroleum-based resins.

In general, it has been evidenced that the properties of the investigated bio-based UPRs can be fine-tuned by the proper selection of glycols in the prepolymer feed. Based on the presented data, isosorbide significantly increases the resin stiffness and, therefore, should be used in lower amounts. On the other hand, neopentyl glycol affects the increase in the resin’s flexibility and elasticity. 

## Figures and Tables

**Table 1:** Activation energy of flow (Ea_η_) at the constant shear rate (10 s^−1^) of examined UPRs.

	UPR-PG	UPR-IS	UPR-NPG	UPR-PG/IS	UPR-PG/NPG	UPR-NPG/IS
*E*a_η_, kJ/mol	65.4	91.9	58.1	77.4	59.1	72.1

**Table 2:** The UPRs gel content and glass transition temperature obtained by DSC (Tg _DSC_) and DMA (Tg _DMA_), storage moduli at 25 °C (G′_25°C_) and 135 °C (G′_135°C_), and cross-link density (ν_e_).

Sample	Gel content, %	T_g DSC_, °C	T_g DMA_, °C	G′_25 °C_, GPa	G′_135 °C_, MPa	ν*_e_*, mol m^−3^
UPR-PG	98.01	65.5	87.0	1.13	9.89±1.48	971.7457
UPR-IS	75.49	70.8	/	/	/	/
UPR-NPG	97.55	66.4	92.0	0.996	9.58	941.2865
UPR-PG/IS	91.21	66.2	88.0	1.34	3.98	391.0564
UPR-PG/NPG	99.97	70.6	96.0	1.08	31.3	3075.393
UPR-NPG/IS	96.28	63.7	92.0	1.35	5.02	493.242

**Table 3:** Tensile properties of the prepared UPRs.

Sample	*E,* GPa	*σ,* MPa	*ε_k_*, %
UPR-PG	1.49 ± 0.06	30.5 ± 1.2	3.61 ± 0.33
UPR-IS	/	/	/
UPR-NPG	1.23 ± 0.06	30.4 ± 1.5	3.99 ± 0.37
UPR-PG/IS	1.74 ± 0.38	12.5 ± 2.7	0.90 ± 0.17
UPR-PG/NPG	1.42 ± 0.07	35.1 ± 1.7	4.39 ± 0.39
UPR-NPG/IS	1.55 ± 0.30	15.8 ± 3.1	1.31 ± 0.21

## Data Availability

Data presented in this study are available on request from the corresponding author.
